# Comparison of SynCAM1/CADM1 PDZ interactions with MUPP1 using mammalian and bacterial pull‐down systems

**DOI:** 10.1002/brb3.1587

**Published:** 2020-02-28

**Authors:** Martina Baliova, Frantisek Jursky

**Affiliations:** ^1^ Laboratory of Neurobiology Institute of Molecular Biology Slovak Academy of Sciences Bratislava Slovakia

**Keywords:** MUPP1, PDZ, protein interaction, pull‐down assay, SynCAM1/CADM1

## Abstract

**Background:**

Synaptic cell adhesion molecule 1 (SynCAM1) also known as cell adhesion molecule 1 (CADM1) is a transmembrane cell adhesion protein that operates in a variety of physiological and pathological cellular contexts, and its interaction with the PDZ signalling protein MUPP1 have been previously implicated in autism spectrum disorder (ASD).

**Methods:**

We used in vitro pull‐down systems based on the bacterial and mammalian extracts to study SynCAM1/CADM1 PDZ interactions with MUPP1 at various conditions.

**Results:**

So far, the investigated interaction of SynCAM1/CADM1 with MUPP1 has been mostly attributed to an unspecified region of MUPP1 PDZ domains 1–5 or exclusively to domain 2, using a yeast two‐hybrid system. We also confirmed the single interaction of native synaptosomal CADM1 with PDZ domain 2. However, in this work, using recombinant proteins overexpressed in bacteria, we found an in vitro pull‐down conditions in which all first five domains and, to a much lesser extent, MUPP1 domains 7 and 11 significantly interacted with the whole C‐terminal domain of SynCAM1/CADM1. These PDZ interactions were confirmed by a pull‐down assay using the last seven amino acids of the SynCAM1/CADM1 PDZ motif and using two fusion partners. Multiple interactions were additionally replicated using the continuous N‐terminal MUPP1 protein fragment, which included first five PDZ domains, containing either intact or mutated domain 2.

**Conclusions:**

We hypothesize that multiple interactions might exist in vivo*,* representing transient low‐affinity interactions or alternative binding sites on MUPP1 when domain 2 is occupied or occluded by the interaction with other ligands. This newly identified interactions extend the potential genetic mutations, possibly affecting SynCAM1/CADM1/MUPP1 function. Possible reasons for the absence of some of the identified CADM1 PDZ interactions in mammalian extracts are discussed.

## Graphical abstract

SynCAM1/CADM1 C‐terminus interacts with several MUPP1 domains in overexpressed bacterial extract, while it interacts solely with MUPP1 domain 2 in mammalian cell extract. Multiple interactions might represent transient low‐affinity interactions, outcompeted in mammalian cell extract with other ligands. Newly identified interactions extent the potential targets of genetic mutations affecting SynCAM1/CADM1/MUPP1 function.

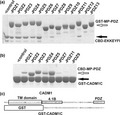

## 1 INTRODUCTION

The members of the cell adhesion molecule (CADM) family are type I transmembrane proteins that have been independently identified by several investigators, and they have acquired several different names, such as *Igsf4* (Ig‐like spermatogenic factor; Gomyo et al., 1999), *TSLC* (tumor suppressor in lung cancer; Kuramochi et al., 2001), *Necl* (Nectin‐like molecules; Takai, Irie, Shimizu, Sakisaka, & Ikeda, 2003), Ra175 (Urase, Soyama, Fujita, & Momoi, 2001), SgIGSF (spermatogenic immunoglobulin superfamily; Wakayama, Ohashi, Mizuno, & Iseki, 2001; Watabe, Ito, Koma, & Kitamura, 2003), and *SynCAM* (synaptic cell adhesion molecule; Biederer et al., 2002). The Human Genome Organization (HUGO) Gene Nomenclature Committee has recently renamed the genes as *CADM* (cell adhesion molecule).

Even though the exact mechanisms by which these adhesion molecules take part in diverse cellular functions remain to be elucidated, CADMs have been implicated in several pathological and physiological processes, such as spermatogenesis (Fujita et al., 2006; Wakayama et al., 2001, 2003; van der Weyden et al., 2006; Yamada et al., 2006), epithelium development and homeostasis (Ito et al., 2007), mast cell adhesion (Furuno et al., 2005; Ito et al., 2003, 2007; Ito & Oonuma, 2006), progression of lung and other cancers (Murakami, 2005), central nervous system development (Biederer et al., 2002; Sara et al., 2005; Spiegel et al., 2007), and autism (Fujita, Tanabe, Imhof, Momoi, & Momoi, 2012). Autism spectrum disorder (ASD) is a highly heritable disorder with altered cognitive ability and fundamental deficits in social reciprocity, without clear definition of the molecular pathogenesis. Two missense mutations, C739A(H246N) and A755C(Y251S), were previously found in the CADM1 gene of male Caucasian ASD patients and their family members (Zhiling et al., 2008). These mutations impaired CADM1 function and altered synaptogenesis in neurons (Fujita et al., 2010), which underlies the possible involvement of CADM1 in certain conditions of ASD pathogenesis.

So far, four CADM genes have been identified in tetrapod vertebrates, possessing identical structural organization. CADM proteins consist of three extracellular Ig‐like loop domains, a transmembrane domain, and a cytoplasmic tail. The cytoplasmic tail is highly conserved and contains two short protein–protein interaction domains, namely a juxta membrane protein 4.1 binding motif (FERM domain binding) and a C‐terminal type II PDZ binding motif (Biederer, 2006).

PDZ binding motifs are short stretches of amino acid residues (<10 amino acids) at the extreme carboxyl termini of proteins, which bind to the groove of specially folded, about 100 amino acid long PDZ domains (Doyle et al., 1996; Songyang et al., 1997). The multiple signalling protein MUPP1 contains 13 such PDZ domains (Ullmer, Schmuck, Figge, & Luëbbert, 1998), and its interaction with CADM1 has been previously reported, with either PDZ domain 2 using a yeast two‐hybrid system (Jang et al., 2014) or with an unspecified region of PDZ domains 1–5, interacting with the GST‐CADM C‐terminal region expressed in mammalian cells (Fujita et al., 2012). Here, we found the interaction condition in which all of the first five domains, individually, and, to a much lesser extent, domains 7 and 11 of MUPP1 showed significant affinity to the bacterially expressed whole C‐terminal domain as well as to the PDZ binding motif of SynCAM1/CADM1. We additionally report the pull‐down of Triton X‐100 solubilized native synaptosomal CADM1 protein interacting solely with MUPP1 domain 2.

## 2 MATERIALS AND METHODS

### 2.1 Chemicals and antibodies

Glutathione Sepharose^®^ 4B and anti‐Glutathione‐S‐Transferase (anti‐GST) antibodies produced in rabbits were purchased from Sigma Chemicals. The human CADM1 polyclonal antibody, against peptide VNKSDDSVIQLLNPNRQTIYFRDFRPLKD, made in rabbits (ThermoSci #PA5‐24196), corresponding to amino acids 66–94 in the extracellular Ig‐like‐V‐type domain of CADM1, was used at a 1:1,000 dilution. The sequence is identical to that of the mouse orthologue. Goat secondary horseradish peroxidase‐conjugated antibodies were purchased from Millipore. Tris (Hydroxymethyl) aminomethane (Tris) freebase, HEPES free acid, and sodium chloride (NaCl), all molecular biology grade, were purchased from Merck Chemicals.

### 2.2 Construction of fusion proteins containing the mouse SynCAM1/CADM1 C‐terminal domain and the CADM PDZ binding motif

Mouse CADM isoform 1 CCDS23149.1. [Q8R5M8‐1] (456 amino acid) was amplified by RT‐PCR from mouse neuroblastoma N2a cell cDNA using forward *Eco*RI 5′‐ctgaattcatggcgagtgctgtgctgccgagcggatcc‐3′ and reverse *Sal*I primer 5′‐tcaggtcgacttagatgaagtactctttcttttcttcggag‐3′. The PCR fragment was purified, digested with *Eco*RI/*Sal*I, and cloned into plasmid pEGFP‐N1 (Clontech/Takara), and the DNA sequence was verified by Sanger DNA sequencing. The DNA coding region of the whole mCADM1 C terminus (amino acids R415‐I456) was obtained by PCR using forward *Eco*RI‐C primer 5′‐ctatgaattcagacataaaggtacatacttcactcatg‐3′ and reverse *Sal*I primer 5′‐tcaggtcgacttagatgaagtactctttcttttcttcggag‐3′. The fragment was purified, digested *Eco*RI/*Sal*I, and inserted in‐frame downstream of the cellulose‐binding domain (CBD) protein‐coding sequence into *Eco*RI/*Sal*I‐digested pET34b (Novagen, Merck), frame‐shifted previously by inserting single cytosine between *Bam*HI and *Eco*RI sites, using site‐directed mutagenesis. Two complementary primers, 5′‐aattcgaaaagaaagagtacttcatctag‐3 and 5′‐tcgactagatgaagtactctttcttttcg‐3 (encoding the last 7 amino acids of the PDZ binding motif of mCADM1 EKKEYFI), and two additional complementary primers, 5′‐aattcgaaaagaaagagtagctgcagg‐3 and 5′‐tcgacctgcagctactctttcttttcg‐3 (coding identical protein region missing last three amino acids YFI), were designed to additionally contain *Eco*RI and *Sal*I restriction sites downstream of the stop codon. The primers were annealed and inserted into *Eco*RI/*Sal*I‐digested pET34b, previously frame‐shifted by inserting a single cytosine between *Bam*HI and *Eco*RI sites using site‐directed mutagenesis. The final constructs contained either the intact, 7‐amino acid‐long PDZ binding region of mCADM1 or its truncated form in‐frame with the CBD protein.

The rat, 156‐amino acid‐long GlyT2 N‐terminal DNA fragment, previously named rGlyT2Nrcf1 (Jursky & Baliova, 2013), was amplified using NdeI 5′‐aattccatatggattgcagtgctcc‐3′ and *Bam*HI 5′‐acagggatcccatattcacccagcccacgg‐3′ primers and inserted into pET21a plasmid (Novagen, Merck) digested with *Nde*I/*Bam*HI. The plasmid was subsequently isolated, opened with *Eco*RI/*Sal*I, and the *Eco*RI/*Sal*I mCADM1 PDZ linker was inserted into these sites, resulting in‐frame fusion of the CADM PDZ motif with GlyT2N terminal protein sequences.

Individual MUPP1 PDZ domains fused to GST were isolated as previously described (Baliova, Juhasova, & Jursky, 2014). To express MUPP1 PDZ domains fused with the CBD protein, the NdeI restriction site in the CBD coding sequence of pET34b was eliminated by site‐directed mutagenesis without changing protein coding. The CBD coding sequence was then transferred to pET28a as the *Nde*I/*Eco*RI PCR fragment in‐frame with the 6X histidine tag. *Eco*RI/*Sal*I fragments of MUPP1 domain coding sequences of MUPP1 (Baliova et al., 2014) were transferred to the pET28a‐CBD plasmid. For the overexpression of the continuous N‐terminal fragment MUPP1, encoding the first five PDZ domains, the DNA sequence was amplified as the *Eco*RI/*Sal*I PCR fragment using primers 5′‐acgaattcacgccaccatgttggaaaccatagacaaaaatcg‐3′ and 5′gagcagtcgacttagtcttccttggctgaaacatac‐3′. Following digestion with *Eco*RI/*Sal*I, the fragment was inserted into the pGEX‐5X‐1 plasmid (GE Healthcare). The final construct started with MUPP1‐initiating methionine and ended with the MUPP1 protein sequence YVSAKED*. To impair PDZ function of PDZ domain 2, the hydrophobic leucine in its carboxylate binding loop GLG protein sequence was replaced with arginine using site‐directed mutagenesis with primers 5′‐gatgggtccggcagagggtttggcatc‐3′ and 5′‐gatgccaaaccctctgccggacccatc‐3′.

### 2.3 Protein overexpression and isolation

All DNA constructs used for protein expression were transformed into *E. coli* BL21 DE3. Cells transformed with GST, CBD, and GlyT2Nrcf1 fusion proteins were cultured overnight to saturation, and following a 1:30 dilution in an appropriate volume of fresh LB cultivation medium, the cells were further cultured for 2 hr at 37°C. GST and CBD cultures were transferred to a 17°C shaker (180 rpm) and cultured for 1 hr in order to cool down. Following the addition of IPTG to 0.3 mM final concentration, cells were shaken overnight at 17°C. Cells were suspended in buffer A (25 mM Tris‐HCl, 150 mM NaCl, 5 mM EDTA, pH 7.5). Triton X‐100 was added to a final concentration of 1%, and proteins were released by sonication. Cell debris was removed by centrifugation. GST fusion proteins were immobilized on glutathione resin and stored at –18°C. CBD‐EKKEYFI and CBD‐EKKE fusion proteins containing cell supernatant extracts were stored at –18°C until use. The GlyT2Nrcf1‐EKKEYFI fusion protein was obtained similarly, except that IPTG induction was performed for 2 hr at 37°C. A small amount of CBD‐EKKEYFI and CBD‐EKKE, used as protein gel running controls, were isolated on microcrystalline cellulose and eluted with SDS running buffer. Since the GlyT2Nrcf1‐EKKEYFI fusion protein is highly flexible (Jursky & Baliova, 2013), a small amount of it was obtained by boiling the extract supernatant for 5 min. Following centrifugation, GlyT2Nrcf1‐EKKEYFI remained in the supernatant, while the majority of the denatured proteins sedimented. The supernatant sample was mixed with SDS running buffer and used as the GlyT2Nrcf1‐EKKEYFI protein gel running control.

### 2.4 Protein interactions

GST‐MUPP1‐PDZ fusion proteins immobilized on resin were thawed, the resin was washed once with interaction buffer, and extracts containing overexpressed CBD‐EKKEYFI, CBD‐EKKE, GlyT2N rcf1‐EKKEYFI, and rcf1‐EKKE proteins were centrifuged 5 min at 13,000 *g*. For the interaction, 30 µl of the resin was mixed with 1 ml of the extract, and the interaction was performed for 1 hr on a rotator at room temperature. The resins were washed 3× with 1 ml of interaction buffer, and fusion proteins were eluted with 20 mM glutathione (pH 8.0) or SDS sample buffer. Following mixing with the SDS sample buffer and a 1‐min boiling step, the samples were resolved on 12% polyacrylamide gel. The gel was stained with Coomassie Brilliant Blue G‐250, and protein bands were quantified by scanning and plotted with GraphPad Prism (GraphPad Software). For investigation of native CADM interactions, crude mouse brain synaptosomes were used (Baliova, Betz, & Jursky, 2004; Baliova & Jursky, 2010). Synaptosomes were solubilized in interaction buffer (25 mM Tris‐HCl, 150 mM NaCl, 1 mM EDTA [pH 7.5], 1% Triton X‐100) and sedimented at 13,000 *g* for 15 min, and potential insoluble aggregates were removed from the supernatants by incubation with a small aliquot of glutathione sepharose resin on the rotator. Following centrifugation, the supernatant interacted for 3 hr at 20°C with MUPP1 PDZ domain GST fusion proteins, isolated as previously described (Baliova et al., 2014). After washing with interaction buffer, glutathione‐eluted interaction complexes were resolved on 7.5% polyacrylamide gel and immunoblotted with anti‐CADM1 antibodies. Fusion proteins were visualized with anti‐GST antibodies.

## 3 RESULTS

**Figure 1:**
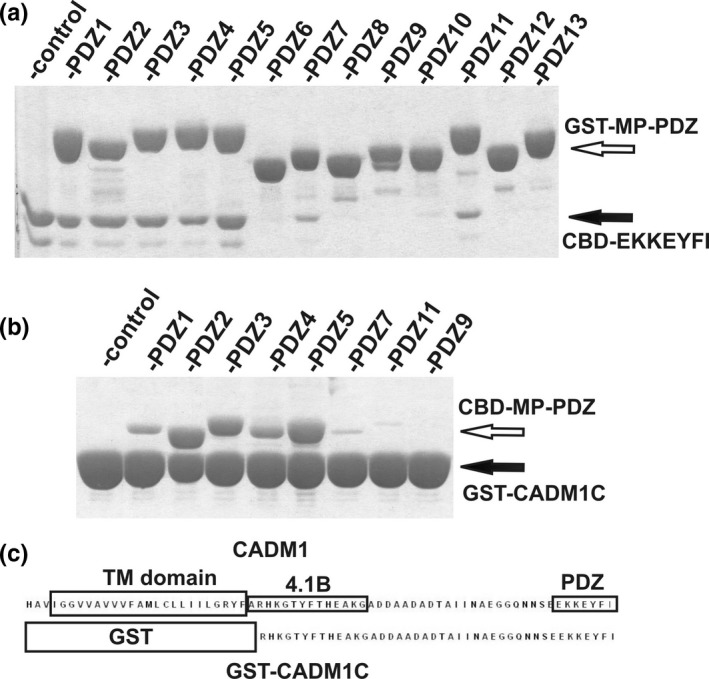
Interaction of the CBD‐EKKEYFI fusion protein containing the CADM1 PDZ motif (EKKEYFI) with GST proteins fused with MUPP1 PDZ domains 1–13 (GST‐MP‐PDZ) (a). Interaction of the GST protein containing the whole cytosolic C‐terminal region of CADM1 (GST‐CADM1C) with the CBD protein fused in‐frame with certain MUPP1 PDZ domains (CBD‐MP‐PDZ) (b). Part c shows the end of the CADM1 protein aligned with the GST‐CADM1C fusion protein used in the pull‐down assay (c). The positions of the transmembrane domain, FERM binding 4.1B domain, PDZ binding motif, and the GST fusion tag are highlighted

To investigate PDZ interactions of MUPP1, we previously used a simple pull‐down system consisting of a GST protein tag fused to all individual 13 PDZ domains of MUPP1 (Baliova et al., 2014). GST fusion proteins were immobilized on glutathione resin and subsequently used to pull down the CBD protein fused to various PDZ binding motifs. Here, we fused CBD with the CADM PDZ binding motif (EKKEYFI) and screened its interactions with all 13 MUPP1 domains. Figure 1a shows that the PDZ motif interacted with the first five domains and, to a lesser extent, with domains 7 and 11. In order to further verify the interaction, we exchanged the GST fusion partner of PDZ domains with the CBD protein and, conversely, the CADM PDZ motif was fused to the GST tag (Figure 1b). Additionally, the whole CADM1 C‐terminal sequence was used instead of the 7‐amino acid‐long PDZ motif (Figure 1c). As shown in Figure 1b, the interaction results were very similar.

**Figure 2:**
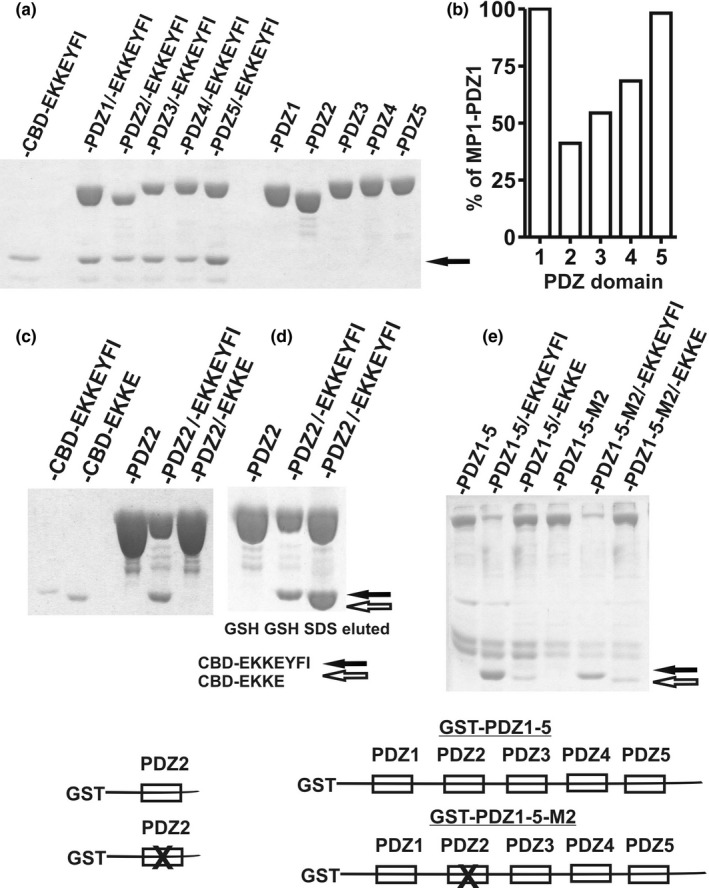
Stabilization of the GST fusion protein interaction with glutathione sepharose with certain PDZ interactions of the CBD‐EKKEYFI protein. The glutathione elution efficiency of GST fusion proteins containing domains 2, 3, and 4 decreased following interaction with the CBD‐EKKEYFI protein (a, left) when compared to the domains that previously interacted with only the interacting buffer (a, right). The relative elution efficiency is quantified in (b). The elution deficiency of domain 2 was absent when the interacting protein CBD‐EKKEYFI was truncated to CBD‐EKKE (c). A comparison of glutathione‐ (GSH) and SDS‐eluted samples suggests that the elution deficiency was caused by the stabilization of the fusion protein on the glutathione resin (d). A similar stabilization effect is observed when the whole N‐terminal domain, including the first five domains of MUPP1 (GST‐PDZ1‐5), is used (e). The effect is dependent on the presence of the ‐YFI PDZ motif. The PDZ interaction and stabilization effect of several domains is supported by its presence in the construct GST‐PDZ1‐5‐M2 with mutated PDZ domain 2. Filled and open arrows indicate the position of CBD‐EKKEYFI and CBD‐EKKE proteins

Figure 1a shows the interaction of GST‐PDZ domains with CBD‐EKKEYFI when eluted fusion proteins were proportionally adjusted to equal amounts of the eluted GST‐PDZ fusion protein. However, when the eluted proteins were not adjusted and compared to the eluted GST‐PDZ proteins previously interacting solely with the interaction buffer, significant differences between the amount of eluted proteins were seen in domains 2, 3, and 4 (Figure 2a,b). To study whether the effect was caused by PDZ interaction motif‐YFI, in addition to the CBD‐EKKEYFI protein, we constructed its deleted variant CBD‐EKKE. Even though the new C‐terminal end of CBD‐EKKE likely decreased its turnover, resulting in a higher amount in the extract (Parsell, Silber, & Sauer, 1990), the amount of eluted GST‐PDZ2 protein did not decrease when compared to the control (Figure 2c). A comparison of glutathione and the SDS‐eluted GST‐PDZ2 protein indicated that the fusion protein was stabilized on the resin by the PDZ interaction rather than being washed off (Figure 2d). A stabilization effect was also seen in the GST fusion protein when the whole N‐terminal domain, including the first five domains of MUPP1 (GST‐PDZ1‐5), was used and when PDZ function of domain 2 was disrupted (Figure 2e). Again, CBD‐EKKE protein missing YFI PDZ motif did not cause this effect (Figure 2e).

**Figure 3:**
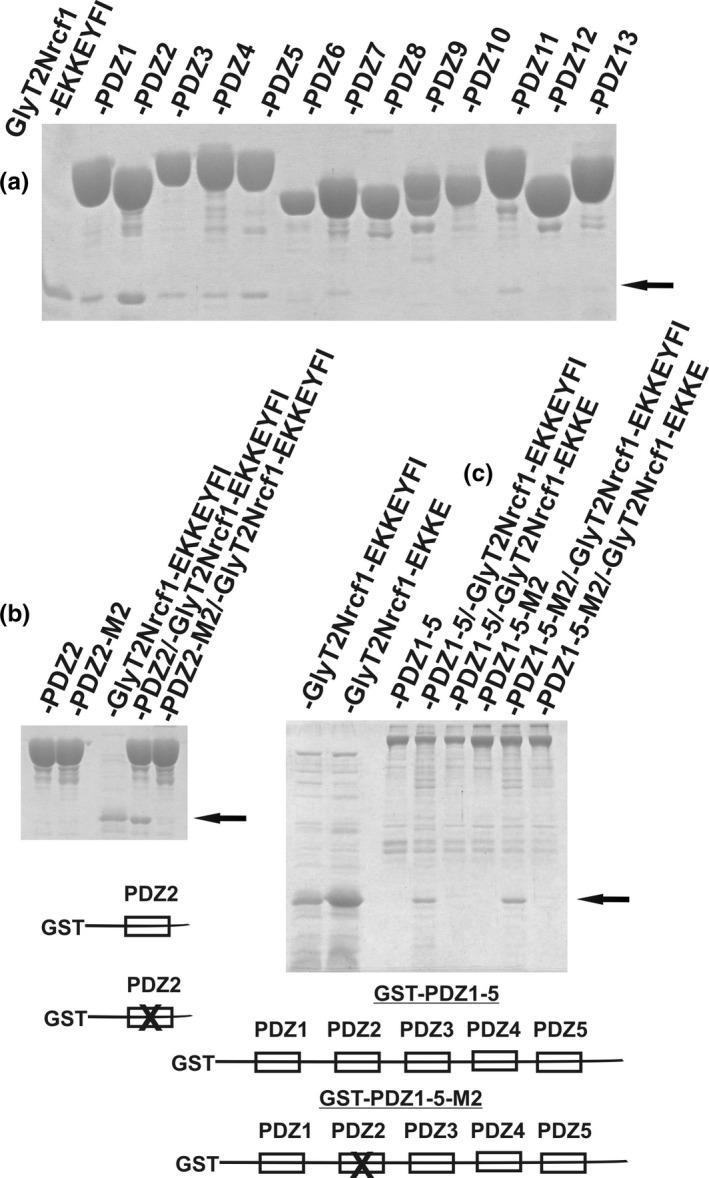
The interaction of the CADM1 PDZ motif containing the protein GlyT2Nrcf1‐EKKEYFI (marked by filled arrow), with GST proteins fused to the MUPP1 PDZ domains (a). The interaction was not observed when the GLG motif in PDZ domain 2 was replaced with GRG (marked as ‐PDZ2‐M2) (b). Similarly, the YFI‐deleted GlyT2Nrcf1‐EKKE protein did not interact when the whole N‐terminal domain, including the first five domains of MUPP1 (GST‐PDZ1‐5), was used for the interaction (c). On the other site, GST‐PDZ1‐5‐M2, whose PDZ2 function was disrupted by GLG‐GRG mutation still interacted with GlyT2Nrcf1‐EKKEYFI protein, supporting its interaction with other domains which is part of the GST‐PDZ1‐5‐M2 protein (c). The filled arrow indicates the position of both GlyT2Nrcf1‐EKKEYFI and GlyT2Nrcf1‐EKKE proteins

**Figure 4:**
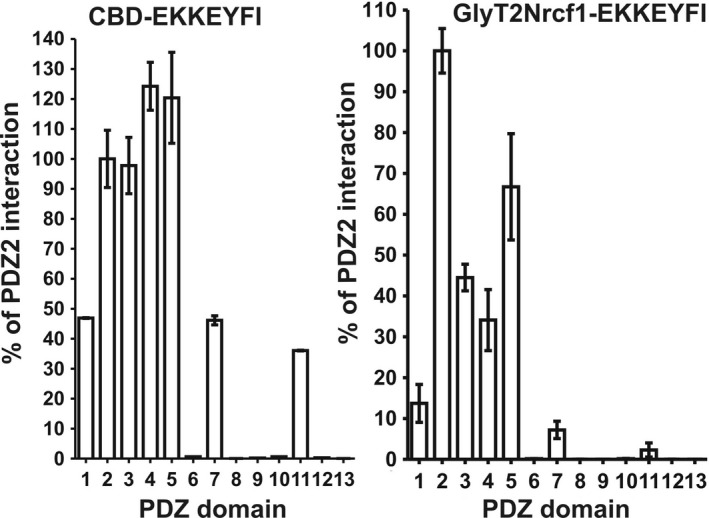
Comparison of the PDZ interaction of the MUPP1 domains with the CADM1 EKKEYFI PDZ motif fused with the CBD protein and the GlyT2Nrcf1 protein. Note that while the specificity of the interaction with MUPP1 domains is identical for both protein fusion partners, the interaction affinity differs significantly when the highly structured CBD fusion partner is replaced by the unstructured GlyT2Nrcf1 fusion partner. The samples were eluted with SDS buffer to prevent the observed effect of different elution efficiencies of glutathione. Data represent the mean ± *SEM* of three independent experiments

The CBD protein is a highly structured protein containing mainly β‐sheets. In order to investigate whether the CBD structure contributes to this effect, we fused the CADM PDZ motif (EKKEYFI) with the mostly disordered N‐terminal protein sequence of glycine transporter GlyT2N (Juhasova, Baliova, & Jursky, 2016), resulting in protein GlyT2Nrcf1‐EKKEYFI. The whole *E. coli* cellular extract containing the overexpressed protein was used for the interaction. To obtain a protein gel running control for GlyT2Nrcf1‐EKKEYFI protein, a heat denaturation procedure was used for its partial purification (Jursky & Baliova, 2013). Figure 3a shows that even though the interaction profile showed identical specificity with CBD‐EKKEYFI (Figure 1a), here, the interaction of domain 2 significantly dominates. As shown in Figure 3b, the interaction of single PDZ domain 2 was not observed when the GLG motif was mutated to GRG. However, when domain 2 was mutated in the GST fusion protein containing the first five MUPP1 PDZ domains, the PDZ interaction was still present, suggesting participation of other PDZ domains (Figure 3c). Figure 3c also shows that, again, the interaction was dependent on the presence of the last three amino acids (‐YFI). The interaction of the truncated GlyT2Nrcf1‐EKKE protein was not observed, despite its much higher abundance in the extract (Figure 3c). However, the results in Figure 3 also show that when the GlyT2N terminal sequence is used as the fusion partner, the interaction is not stabilized to the extent that it is with the CBD fusion tag, and no stabilization of the GST protein on the glutathione resin is observed. Figure 4 shows the comparison of the PDZ interaction of the CADM1 EKKEYFI PDZ motif fused with the CBD protein and the GlyT2Nrcf1 protein to MUPP1 domains. While the specificity of the interaction with MUPP1 domains is identical for both protein fusion partners, the interaction affinity differs significantly when the highly structured (CBD) fusion partner is replaced with the unstructured GlyT2Nrcf1 fusion partner. The samples were eluted with SDS buffer to prevent the observed effect of different elution efficiencies of glutathione.

**Figure 5:**
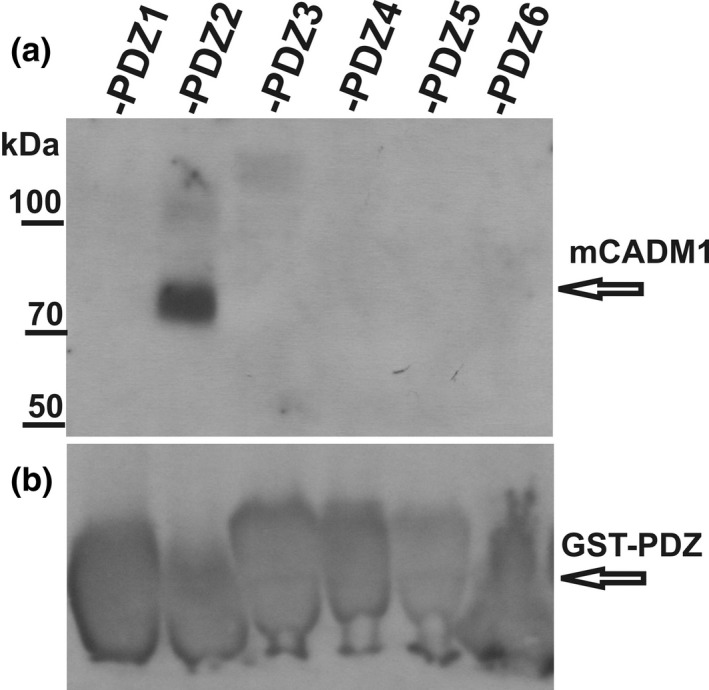
Interaction of Triton X‐100‐solubilized synaptosomal CADM1 with MUPP1 PDZ domains 1–6. GST fusion proteins fused in‐frame with PDZ domains 1–6 immobilized on glutathione resin interacted with the Triton X‐100 extract of mouse synaptosomes. Following washing, interaction complexes were eluted with 20 mM glutathione, resolved on 7.5% polyacrylamide gel, and immunoblotted with anti‐CADM1 antibodies (a). GST fusion proteins were visualized with anti‐GST antibodies (b)

To see which PDZ domains would pull down native CADM1 from cellular extracts, we prepared a Triton X‐100‐solubilized extract from mouse synaptosomes and interacted it with MUPP1 domains (Figure 5). The GST fusion proteins fused in‐frame with individual MUPP1 PDZ domains 1–6 interacted with the synaptosomal extract, and following the elution of interaction complexes with glutathione, they were resolved on 7.5% acrylamide gel. The position of CADM1 (about 70 kDa protein) was identified by immunoblot using anti‐CADM1 antibodies. In contrast to the interaction obtained with *E. coli* lysate, in mammalian cell extracts the results showed the exclusive interaction of PDZ domains 2, and no significant interaction was detected with domains 1, 3, 4, 5, and 6.

## 4 DISCUSSION

While the interaction of MUPP1 with its interaction partners can be verified using coimmunoprecipitation of intact interacting proteins, investigation of the interaction specificity of individual MUPP1 domains is impossible without their dissection using the methods of molecular cloning. In such an approach, the PDZ protein sequence is usually coupled to a recombinant fusion partner for efficient bacterial expression and affinity immobilization on carriers for purposes of a pull‐down assay. Fusion partners and the dissected protein domain region together introduce unpredictable steric constraints, which might cause both false reactivity as well as no reactivity, leading to possibly different results obtained by investigators. The interaction of CADM1 with MUPP1 has previously been studied by the pull‐down technique using the GST‐CADM C‐terminal region expressed in mammalian cells, and these experiments limited the interaction to the unspecified region of MUPP1 PDZ domains 1–5 (Fujita et al., 2012). A later study exploited a yeast two‐hybrid system, and the results suggested the exclusive participation of MUPP1 PDZ domain 2 in the interaction with the CADM1 PDZ motif (Jang et al., 2014). We previously developed a simple PDZ interaction assay system, allowing for the study of PDZ interactions for all 13 MUPP1 PDZ domains (Baliova et al., 2014). When we adapted this assay to probe the PDZ interaction of the CADM1 PDZ motif with MUPP1 domains, we detected a positive interaction of all of the first five domains and weaker interactions with domains 7 and 11. These interactions were present regardless of whether we used the short, 7‐amino acid PDZ binding motif or the whole CADM1 C‐terminal region. The interactions were also preserved when fusion protein tags of interacting partners were exchanged. Additionally, when the whole N‐terminal region of MUPP1, including the first five domains of MUPP1, was fused with GST (GST‐PDZ1‐5), and the PDZ interaction was still observed after the PDZ function of domain 2 was disrupted. This clearly indicates that in addition to domain 2 other domains contribute to CADM1 PDZ interactions with MUPP1.

During our experiments, we observed a significant difference between the stabilization of PDZ interactions with highly ordered CBD versus mostly disordered GlyT2Nrcf1 fusion protein partners. This suggests that upstream sequences affected the strength but not the specificity of the interaction. The PDZ nature of the interaction was confirmed by its elimination following deletion of the last three amino acids, which are essential for PDZ interactions.

Interestingly, some of the CBD‐PDZ interactions increased the affinity of the GST‐PDZ domains for the glutathione resin. Even though stabilization was dependent on the CADM1 PDZ motif, it also required the presence of the CBD protein moiety. The effect was observed with GST fused to single PDZ domain 2, as well as the first five domains of MUPP1 (GST‐PDZ1‐5), which excludes identical steric constraints. Because CBD‐EKKEYFI proteins do not bind to glutathione resin (not shown), it is more likely that a weak interaction of the CBD moiety with the GST protein might partially occlude the access of glutathione.

In contrast to the pull‐down assay using *E. coli* extract, in the Triton X‐100‐solubilized synaptosomal extract when the intact CADM1 protein was pulled‐down, only the interaction of MUPP1 PDZ domain 2 was confirmed using the GST‐PDZ2 fusion protein. Two major reasons can be considered when explaining the absence of an interaction in some domains in mammalian extracts containing the native CADM1 protein. The first reason might be the steric collision of an artificially introduced GST fusion tag with the intact cellular CADM1 glycosylated protein moiety (which is normally separated from the PDZ motif by a membrane). The second might be the possible competition interference of endogenous ligands. In contrast to mammalian cells extracts in bacterial systems, PDZ interactions are rare since bacterial “PDZ‐like” domains are significantly different from canonical metazoan PDZ domains (Ponting, 1997). Additionally, in homogenized cellular extracts the protein interaction conditions might be very different from that existing in living cells in vivo. In living cells, trafficking protein complexes are too bulky and do not diffuse freely. Molecular motors are required for active protein transport along microtubules or actin tracks into separated cellular compartments (Setou, Nakagawa, Seog, & Hirokawa, 2000). In contrast to homogenized cellular extracts, here, unwanted ligands are insulated and excluded from competition and interference. The similarity of the interaction profile between CADM1 and the GABA transporter GAT1 PDZ motif, reported previously (Baliova et al., 2014), indicates that other ligands might indeed exist and cause hidden interference, resulting in false non‐reactivity of ligands with PDZ domains in homogenized extracts of cells or tissues. In addition to binding to carboxyl peptides, some of the PDZ domains can also interact with internal peptide fragments randomly present in extracts (Hillier, Christopherson, Prehoda, Bredt, & Lim, 1999). Competition with CADM1 PDZ motif might even cause small molecules (Fang et al., 2003; Fujita et al., 2006; Grillo‐Bosch, Choquet, & Sainlos, 2013; Tenno et al., 2013) and randomly ordered or disordered peptides with highly frequent interaction on/off rates, which cannot be physically recovered by a pull‐down assay (Fang et al., 2003; Grillo‐Bosch et al., 2013; Piserchio et al., 2004; Wiedemann et al., 2004).

In overexpressed bacterial extracts, another issue should be considered, such as the high concentration of overexpressed ligand, potentially leading to false interactions of low‐affinity PDZ ligands, which might not be physiologically significant in vivo*.*

In our case, however, the CADM1 interaction with several PDZ domains had an interaction magnitude comparable to the already established interaction of domain 2. No interaction was observed following deletion of the PDZ motif, which indicates that in bacterial extracts a high concentration of ligand allowed shorter incubation times to reach interaction equilibrium, without changing the ligand specificity. Additionally, during the trafficking and interaction in vivo, PDZ ligands are likely directly juxtaposed, without concentration equilibration with the bulk solution. This rather resembles the conditions of highly frequent availability of ligand, similarly as it is in conditions of pull‐down assay using bacterially overexpressed ligands.

## 5 CONCLUSIONS

Creation of the MUPP1 signalling complex may proceed via concatenation of ligands on a single molecule when the ligands occupy different PDZ domains or by clustering of MUPP1 molecules when the ligands interact with the identical MUPP1 PDZ domain. The advantage of the CADM1 interaction with multiple MUPP1 domains reported here could be the possibility of interaction with an alternative domain, when some domains are occupied with other ligands, or they are for some reasons sterically occluded. Finally, multiple low‐affinity interactions might serve as transient interaction contacts before the ligand reaches the final interaction configuration. The newly identified interaction might additionally extend the potential genetic mutations affecting SynCAM1/CADM1/MUPP1 function.

## CONFLICTS OF INTEREST

The authors declare no conflicts of interest.

## AUTHOR CONTRIBUTIONS

Both authors conceived the study, collected the data, contributed to data interpretation, manuscript writing, and revisions.

## ETHICAL APPROVAL

This article does not contain any studies involving animals or humans performed by any of the authors.

## Data Availability

The data that support the findings of this study are available from the corresponding author upon reasonable request.
